# Reconfigurable Integrated Thermo-Optics for Aberration Correction

**DOI:** 10.1021/acsphotonics.4c01290

**Published:** 2024-10-08

**Authors:** Josep M. Panadés, Nadja Rutz, Hadrien M. L. Robert, Raphael T. Steffen, Jose García-Guirado, Gilles Tessier, Romain Quidant, Pascal Berto

**Affiliations:** † Sorbonne Université, CNRS UMR7210, INSERM UMRS968, Institut de la Vision, Paris 75012, France; ‡ Nanophotonic Systems Laboratory, Department of Mechanical and Process Engineering, ETH Zürich, 8092 Zürich, Switzerland; § Universite Paris Descartes, Sorbonne Paris Cite, Paris 75006, France; ∥ Institut Universitaire de France (IUF), Paris 75005, France

**Keywords:** wavefront shaping, micro-optics, planar optics, adaptive optics, thermal lenses

## Abstract

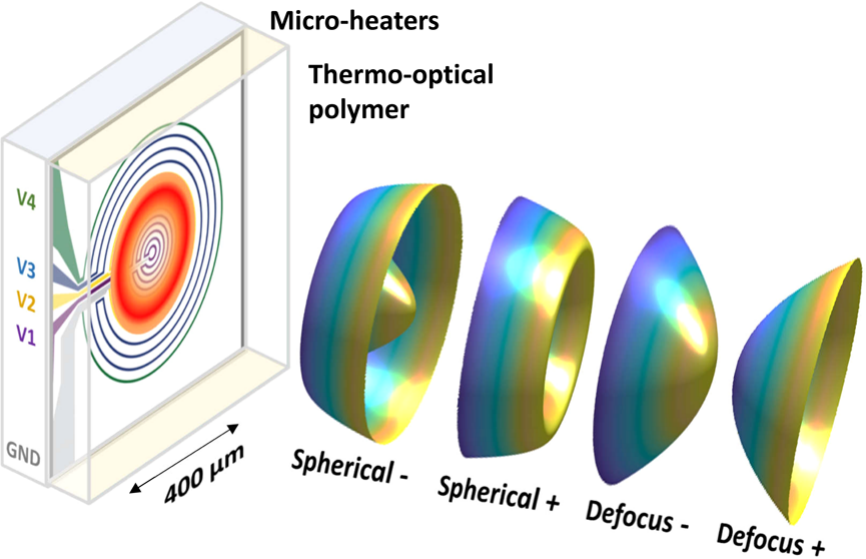

As miniaturization becomes a growing trend in optical systems, the ability to precisely manipulate wavefronts within micrometric pupils becomes crucial. Extensive efforts to develop integrated micro-optics primarily led to tunable microlenses. Among these approaches, *SmartLenses*, which use predesigned microheaters to locally change the refractive index in a transparent thermo-optical material, allow to produce tunable micro-optics with free-form shape. However, the shape and sign of the generated wavefront profile are fixed, predetermined by the geometry of the resistor, which severely limits its use, e.g., for aberration correction. Here, we report a precise reconfigurability of the generated wavefront through dynamic shaping of the temperature distribution, enabled by an independent control of concentric resistors. As a proof of principle, we demonstrate a bimodal *SmartLens* that simultaneously acts as a converging/diverging lens and a positive/negative spherical aberration corrector. Through independent control of Zernike modes, this approach paves the way for compact, broadband, transparent and polarization-insensitive wavefront shapers, with a broad range of potential applications, from endoscopy to information technology.

## Introduction

Photonics has entered a new era, driven by innovative methods for the dynamic manipulation of wavefronts.^1,2^ These advancements enable the real-time modification of the transfer function of optical systems, becoming a key element for active and adaptive optics.^3^ Originally developed to improve astronomical imaging,^4^ aberration correction through adaptive optics is nowadays becoming essential in many fields, including ophthalmology, laser sciences, photolithography, and microscopy.^3,5,6^ Most implementations rely either on the use of deformable mirrors (DM)—for rapid aberration correction—or liquid-crystal spatial light modulators (SLM)—to access a large number of modes.^3^ Since both devices usually operate in reflection mode, integrating them into an optical system demands that the optical path be folded, which increases weight, volume, complexity, and cost. This has recently fostered the development of adaptive lenses (or deformable plates) operating in transmission, which utilize multiple actuators to mechanically deform an interface and correct a few low-order Zernike modes.^7−10^ Despite their proven effectiveness in ophthalmology and microscopy, the volume of these lenses forbids their integration into miniaturized devices.

Nevertheless, miniaturizing optical systems is crucial for a wide range of applications in the visible spectrum, from research and clinical environments to consumer applications. In conjunction with the decreasing size of electronic devices, which has led to e.g., camera sensors with submicron pixels, the reduction in size of optical components has become essential for the development of e.g., portable devices (Smartphones, cameras···), endoscopes^11^ or miniaturized, wearable microscopes.^12,13^ The use of microlens arrays has also become crucial for three-dimensional (3D) displays, 3D light field imaging and ophthalmology.^14^ This has fostered significant advances in optical manufacturing, notably through the development of metasurfaces, which utilize two-dimensional arrangements of meta-atoms to achieve planar optics,^15,16^ or through the use of 3D nanophotolithography, to design freeform, complex micro-optics such as micro-objectives.^17^ In the meantime, the need for *in-operando* focus adjustment has driven the development of adjustable microlenses using e.g., electromagnetic,^18,19^ electromechanic,^20−22^ thermopneumatic,^23,24^ thermal,^25,26^ optical^27^ and liquid-crystal^28^ based technologies. However, microscale wavefront manipulation remains very limited compared to larger systems. Current methods are mainly restricted to spherical wavefront modifications,—i.e. tunable converging or diverging lenses—and do not provide versatile, reconfigurable wavefront control, for e.g., further aberration correction. Metalenses are an interesting candidate as they can now provide some degree of reconfigurability using e.g., electrical or mechanical actuation,^16,29^ but despite recent progresses, most implementations remain complex to manufacture and/or still suffer from diffraction losses and/or operate in relatively narrow bands in the visible range.

In this context, thermo-optic actuation represents a particularly promising approach for micrometer-scale optical wavefront control. It involves inducing local phase shifts by exploiting thermally induced refractive index changes  in a transparent material with a high thermo-optic coefficient . For instance, Polydimethylsiloxane (PDMS,^30^ = −4.5 × 10^–4^ K^–1^), with its relatively high ceiling temperature (*T*_c_ = 250 °C) allows a maximum refractive index modulation of the order of Δ*n*(*T*_c_) ≈ −10^–2^ to 10^–1^. Initial developments relied on optical heating to engineer the local temperature changes Δ*T*, but this typically requires cumbersome systems and lasers.^31^ This prompted the use of engineered electrical resistors instead, microsystems coined as *SmartLenses*,^32,33^ which provide compact, polarization-insensitive devices with smooth, nondiffracting index profiles. The electrical power supplied to the resistors of these initial *SmartLenses* can easily tune the amplitude of the thermally induced phase shift. However, the microfabricated resistor defines a fixed phase modification profile based on the induced temperature distribution and resulting index change. This restricts the ability to dynamically modify the optical function or perform adaptive tuning of multiple Zernike modes for aberration correction. Furthermore, as local cooling at the microscale proves difficult, it is currently not possible to change the sign of a given Zernike mode.

This paper presents a strategy for reconfigurable *Smartlenses* that surpasses simple tunability. These lenses can not only adjust the phase-shift magnitude of the wavefront but also invert the sign of its curvature and modify its overall shape. The method employs a set of independently addressable microresistors to reconfigure the temperature distributions within a thermoresponsive polymer. By activating and balancing specific resistors, we generate and manipulate various wavefront profiles across a submillimeter pupil, with precise control over both the magnitude and shape of the phase modification. As a demonstrator, we designed and tested a 400 μm bimodal *SmartLens*, optimized to provide a full, independent control of two specific Zernike modes, namely defocus and spherical aberration. This approach promises to benefit the development of the next generation of phase control devices, offering significant advantages in terms of broadband operation, polarization insensitivity, high transparency, and simple fabrication.

## Results

**Figure 1:**
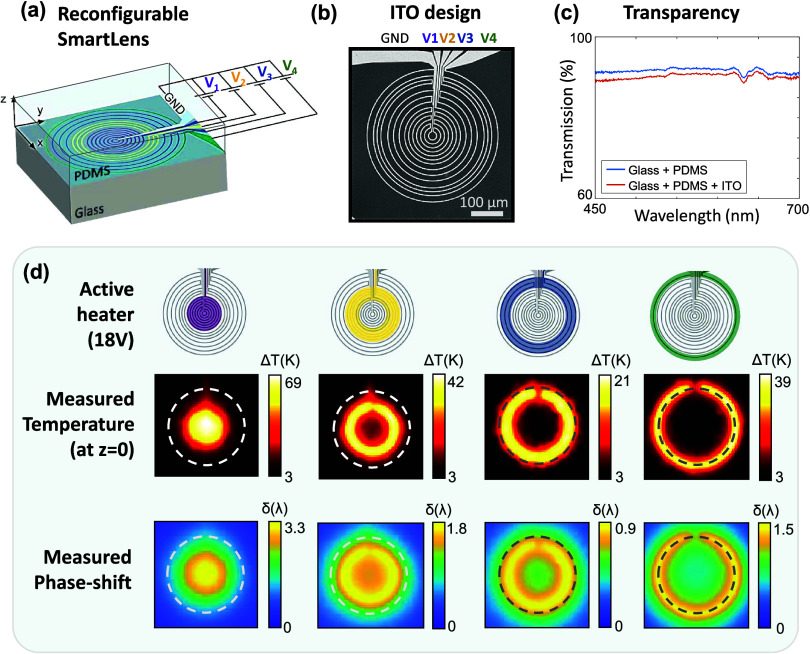
Principle of the electrically reconfigurable *SmartLens*. (a) Schematic: multiple microresistors, each composed of several wire loops (shown in purple, yellow, blue, green), can be independently addressed (V_1_, V_2_, V_3_, V_4_) to create, through Joule effect, various temperature distributions within a thermoresponsive polymer (PDMS) atop the structure. Each independent resistor allows for the control of various refractive index distributions, enabling the dynamic reshaping of the wavefront in transmission. (b) Optical reflection image. (c) Transmission spectrum (red curve). *T*(λ) > 85% in the visible range approaches what is obtained with a similar stack (PDMS + Glass) in the absence of ITO. (d) Thermal responses Δ*T* and optical path difference δ (in units of wavelength, λ) measured at λ = 605 nm when each microresistor is individually biased at 18 V. Dashed lines mark the considered micro pupil size, imposed by the 400 μm diameter of the resistive assembly.

Figure 1a provides a simplified illustration of the reconfigurable *SmartLenses* principle, and Figure 1b shows an optical image of the fabricated resistors, produced using a simple, standard ultraviolet (UV) lithography (see Materials and Methods section). This device consists of several independent microresistive groups of wire loops represented in several colors in Figure 1. These four resistors are individually controlled (see V_1_, V_2_, V_3_, V_4_ in Figure 1a,b) to create, through Joule effect, chosen temperature distributions within a thermoresponsive polymer (PDMS) located on the top (see Figure 1a). The temperature field locally affects the refractive index distribution via the thermo-optical effect. Feeding one or several resistors with chosen voltages thus allows the manipulation of the temperature distribution in PDMS and, therefore, the dynamic reshaping of the transmitted wavefront. In order to address optical modes with rotational symmetry, the microheaters used here are circular loops (except the electrical feeds) but other shapes are of course possible to obtain nonsymmetrical or freeform wavefront modifications. Figure 1d shows the individual thermal (center) and phase (bottom) response of each microheater (top) when applying a mean voltage of 18 V using dedicated electronics (see Materials and Methods section). In each case, the thermally induced optical path difference (OPD) was measured by illuminating the device in transmission (at λ = 605 nm) and imaging the *SmartLens* plane on a high-resolution wavefront sensor. This OPD results from the integration of temperature-induced refractive index changes along the propagation of the optical wave in PDMS. The temperature in the plane of the *SmartLenses* can therefore be deduced from the OPD map using a deconvolution procedure^34^ (see Materials and Methods section). The dotted line delineates the area of the resistors, which can be considered as the pupil size. The measured OPD maps (Figure 1d, bottom) clearly show that multiple wavefront shapes can be generated within a *R*_pup_ ≈ 200 μm radius pupil. The use of Indium tin oxide (ITO), which is relatively transparent in the visible range efficiently mitigates unwanted diffraction effects and allows transmissions exceeding 85%, as shown in Figure 1c. Consequently, this planar, polarization-insensitive, and broadband thermo-optic device can provide an efficient and straightforward method to correct aberrations in miniaturized optical systems.

To illustrate the possibilities of this approach, we chose to control two specific Zernike modes: defocus *Z*_2_^0^ and spherical aberration *Z*_4_^0^. While accessing defocus is clearly essential to e.g., image a selected plane of interest, correcting spherical aberration is also critical in many scenarios to preserve contrast and spatial resolution. Spherical aberration is indeed critical whenever light passes through a slab of material with a mismatched refractive index^35^ (e.g., a glass slide). This situation is extremely common in microscopy, and objective manufacturers designed corrections of the spherical aberration for fixed (e.g., 170 μm glass coverslip correction) or adjustable glass thicknesses. The design shown in Figure 1 has thus been optimized to generate Zernike polynomials related to defocus (*Z*_2_^0^) and spherical aberration (*Z*_4_^0^), both with tunable amplitudes
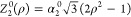
1
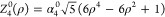
2with ρ the radial distance normalized by the pupil radius *R*_pup_ (ρ = *r*/*R*_pup_), and α_2_^0^ (respectively α_4_^0^) the root-mean-square (RMS) phase value for Defocus (respectively Spherical Aberration). In the following, we detail two key aspects necessary for accessing these pure Zernike modes: the optimization of the resistors design, and the precise determination of the voltage combinations to be applied to each resistor.

**Figure 2:**
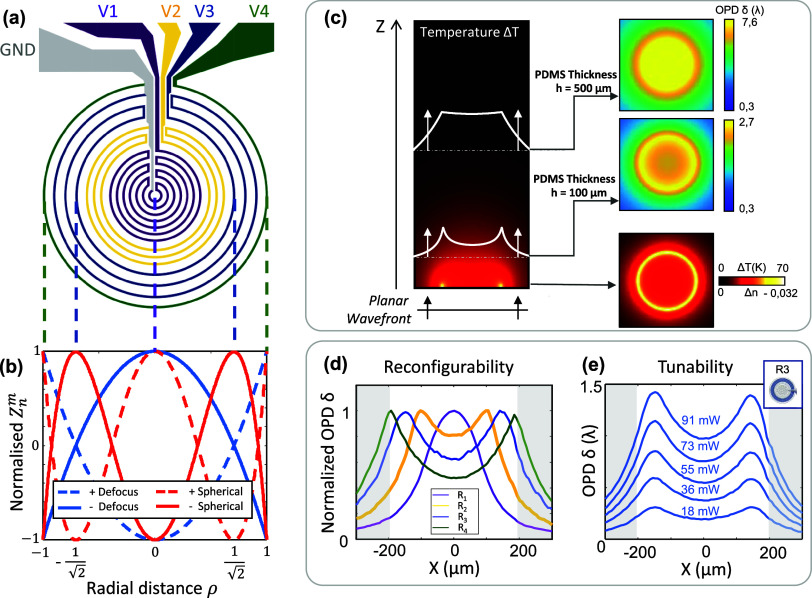
Optimization of the reconfigurable *SmartLens* design. (a) Electrical design optimized to generate Defocus and Spherical aberration using 4 independent microresistors. (b) Radial profile related to Defocus *Z*_2_^0^(ρ) (in blue) and spherical aberration *Z*_4_^0^(ρ) (in red), for positive (dashed line) and negative modes (continuous line). The radius of the largest resistor *R*_4_ (shown in green in (a)) determines the pupil radius, and the relative positions of resistors *R*_1_ (purple) and *R*_3_ (blue) is chosen to match the position of the OPD maxima (ρ = 0, , 1) of the targeted Zernike modes (see vertical dashed lines between (a, b)). (c) PDMS thickness optimization: Numerical simulation of the temperature Δ*T* and OPD maps for different PDMS thicknesses (*h* = 100 and 500 μm). Note the dual colorbar (bottom right) indicating both the temperature rise Δ*T* and the corresponding refractive index change , with  = −4.5 × 10^–4^ K^–1^. A decrease in PDMS thickness leads to a better spatial resolution, although at the expense of a lower phase range. The optimal PDMS thickness therefore results from a trade-off between lateral accuracy and accessible range. (d) Reconfigurability: normalized OPD responses when individually activating resistors *R*_1_ to *R*_4_. (e) Tunability: OPD measured when activating resistor R_3_ with various dissipated power values, in units of the wavelength λ.

The optimized electrical design is shown in Figure 2a, alongside Figure 2b which shows the two targeted Zernike polynomials (described by eqs 1 and 2). The position of the 4 groups of resistive loops has been mainly imposed by considering that a local heating produces a local phase shift. Therefore, the largest resistor *R*_4_ defines the maximum size of the pupil, and the position of the microresistors 1 and 3 was set according to the relative position of the extrema (ρ = 0, 1/√2) of the targeted Zernike modes (see vertical dashed lines between (a) and (b)). The microresistor *R*_2_ provides an additional degree of freedom for negative-coefficient Zernike modes (α_2_^0^ < 0 and α_4_^0^ < 0). Generating positive modes is more difficult, as local heating can only decrease the refractive index and increase the OPD. Since local cooling is not achievable with this resistive design, the refractive index cannot be locally increased, and positive curvatures of the wavefront can only be obtained by heating neighboring regions. The negative spherical mode (α_2_^0^ < 0) has a maximum at a fraction of the pupil radius, which determines the position of resistor *R*_3_, at (see vertical blue dashed lines). Conversely, the positive spherical mode (α_2_^0^ > 0) displays maxima at ρ = 0 and 1, which imposes the position of resistors at the center (*R*_1_) and at the edge (*R*_4_). For each of the four resistors, the wire width and the number of wire loops, from 1 to 7, were fixed in order to result in similar overall resistance values (*R*_1_ to *R*_4_ are typically in the 10 kΩ range). This simplifies the design of the power supply drivers and ensures adequate power dissipation at moderate voltages, typically around 25 V.

The thickness *h* of the thermo-optical material is another crucial parameter, especially for achieving positive Zernike coefficients. To illustrate its influence, let us consider the OPD accumulated as a plane wave propagates through the *SmartLens*. For a given 3D temperature distribution Δ*T*(*r*,*z*), the refractive index varies as  for materials exhibiting linear thermo-optical behaviors. Therefore, the OPD corresponds to the integral of the refractive index change along the propagation direction *z*: 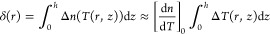
Figure 2c shows, for a given temperature distribution Δ*T*(*r*,*z*), the resulting OPD maps and profiles obtained for two different PDMS thicknesses (*h* = 100 μm and *h* = 500 μm). Here, for the sake of clarity, we considered the temperature distribution induced by a single resistive loop. We can clearly see sharp features (high spatial frequencies) close to the resistor, and broader features (low spatial frequencies) as well as larger OPD values for longer propagation distances *z*. Thinner PDMS therefore allows to preserve high frequency contents, at the expense, however, of the achievable phase range. Here, the PDMS thickness *h* = 230 μm was chosen (see Materials and Methods section) in order to allow convex OPD profiles (see Figure 2d) while keeping a relatively broad dynamic range (see Figure 2e).

Using an appropriate combination of voltages on the various thermal actuators, pure Zernike modes of chosen magnitude can be obtained. To achieve this, let us first describe the response of a given resistor *R*_*i*_. The thermally induced OPD can be estimated by convolving the heat source density map HSD_*i*_(*r*), which represents the power dissipated by Joule effect in resistor *R_i_*, with the OPD Green’s function^33,36^
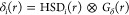
3with , the OPD distribution generated by a point source of heat in a medium of thickness *h*, and κ the average thermal conductivity at the interface between glass and PDMS. By introducing the HSD normalized on the *SmartLens* surface *S*, , we can write
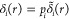
4with  the normalized OPD profile, and *p*_*i*_ the power dissipated by the resistor *R_i_*. This linearity of the OPD response (see eq 4) was verified experimentally over a broad power range (up to 190 mW, see Supporting Section S1). Let us now consider several thermal actuators (i.e., resistors) activated simultaneously. Supporting Section S2 demonstrates that, with a careful design of the ground electrode, the overall response can be assumed to be the sum of the responses from each independently activated thermal actuators:
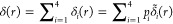
5

This simple linear relation allows, from the individual recorded OPD images δ_*i*_ (see Figures 1d and 2d), to estimate the optimal power combination to apply to each resistor *R*_*i*_ in order to obtain a targeted wavefront δ_target_(*r*). This amounts to solving a non-negative linear least-squares problem of the form:
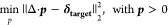
6with Δ *a* matrix built from the concatenation of the **δ̃**_***i***_ vectors, **δ**_**target**_ the vector related to the targeted wavefront, and ***p*** = {*p*_1_,*p*_2_,*p*_3_,*p*_4_} the sought vector giving the optimal r relative power weights. Note that in practice, a fifth parameter *p*_5_ associated with a uniform phase piston, δ̃_5_ = 1, must be included in the least-squares problem to account for the fact that phase offset, or phase piston, is an additional free parameter which is not measurable by wavefront sensing.

**Figure 3:**
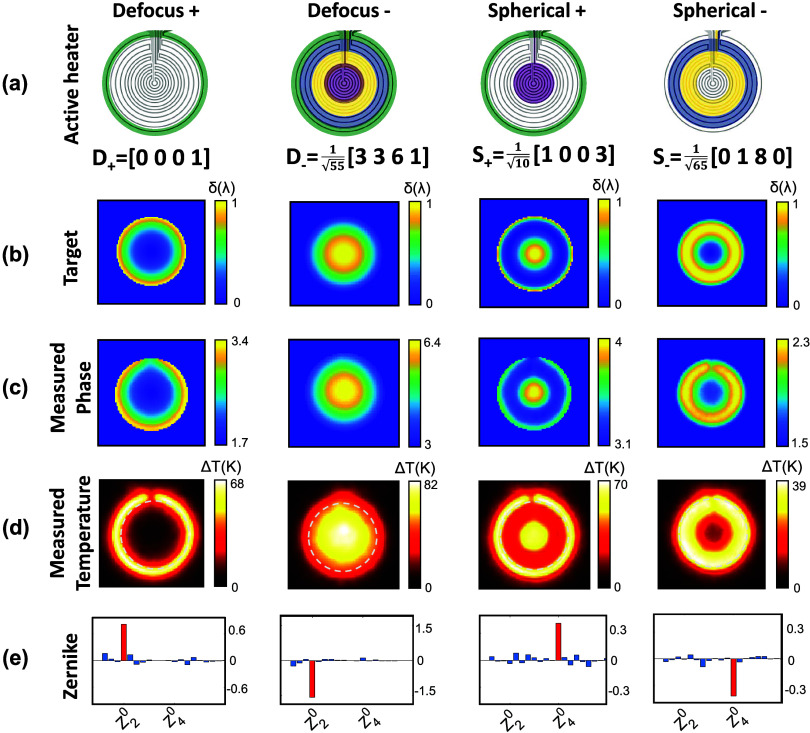
Single reconfigurable *SmartLens* system allows to generate both Defocus and Spherical modes at the microscale. (a) Resistors activated (colors) to achieve targeted wavefronts. The normalized vectors *D*_+_, *D*_–_, *S*_+_, *S*_–_ indicate the relative power weights [*p*_1_*p*_2_*p*_3_*p*_4_] applied to each of the resistors *R*_1_, *R*_2_, *R*_3_ and *R*_4_ in order to obtain the targeted wavefront. (b) Targeted wavefronts. (c) Wavefront measured when applying the optimal voltage combination, showing an excellent agreement with the targeted wavefronts. The results are displayed within the *SmartLens* pupil. (d) Experimental temperature maps (at *z* = 0) deduced from the wavefront maps. The white circle indicates the *SmartLens* pupil. (e) Zernike decomposition of the measured wavefronts (displayed in c). Revealing, in each case, a clear peak corresponding to the targeted Zernike mode (red), with small residue in the other modes (blue).

Figure 3a shows the power combination [*p*_1_*p*_2_*p*_3_*p*_4_] resulting from this least-squares optimization, for each targeted phase map shown in Figure 3b. Here, the vectors ***C*** = ***D***_+_ and ***S***_+_ (respectively ***C*** = ***D***_–_ and ***S***_–_) are normalized vectors related to positive (respectively negative) defocus and spherical aberration, defined as ***C*** = ***p***/*P*_tot_, with *P*_tot_ the total power applied on the reconfigurable *SmartLens* (*P*_tot_ = ∑_i=1_^4^*p*_*i*_). As expected, a positive defocus only requires the outer resistor *R*_4_ to be activated, while a negative defocus requires the activation of all resistors since the PDMS thermo-optical coefficient is negative. Moreover, the result of the least-squares optimization shows that complementary resistors need to be activated to generate either negative or positive spherical aberration, which was also expected. Figure 3c,d show the measured wavefront and temperature maps, obtained when applying these optimal power combinations to the reconfigurable *SmartLens*. In all four cases, the measured OPD maps are in excellent agreement with the targeted wavefronts (see Figure 3b), as quantitatively verified in Figure 3e which shows the Zernike decomposition of the measured wavefronts. In each case, a distinct peak corresponding to the targeted Zernike mode is observed, with minor contributions from other modes. These mostly result from a slight disruption of symmetry in the OPD map caused by the feeding electrodes, as visible in Figure 3c. Although not detrimental, this issue could be mitigated by employing e.g., higher spatial resolution fabrication or multilayer lithography processes.

**Figure 4:**
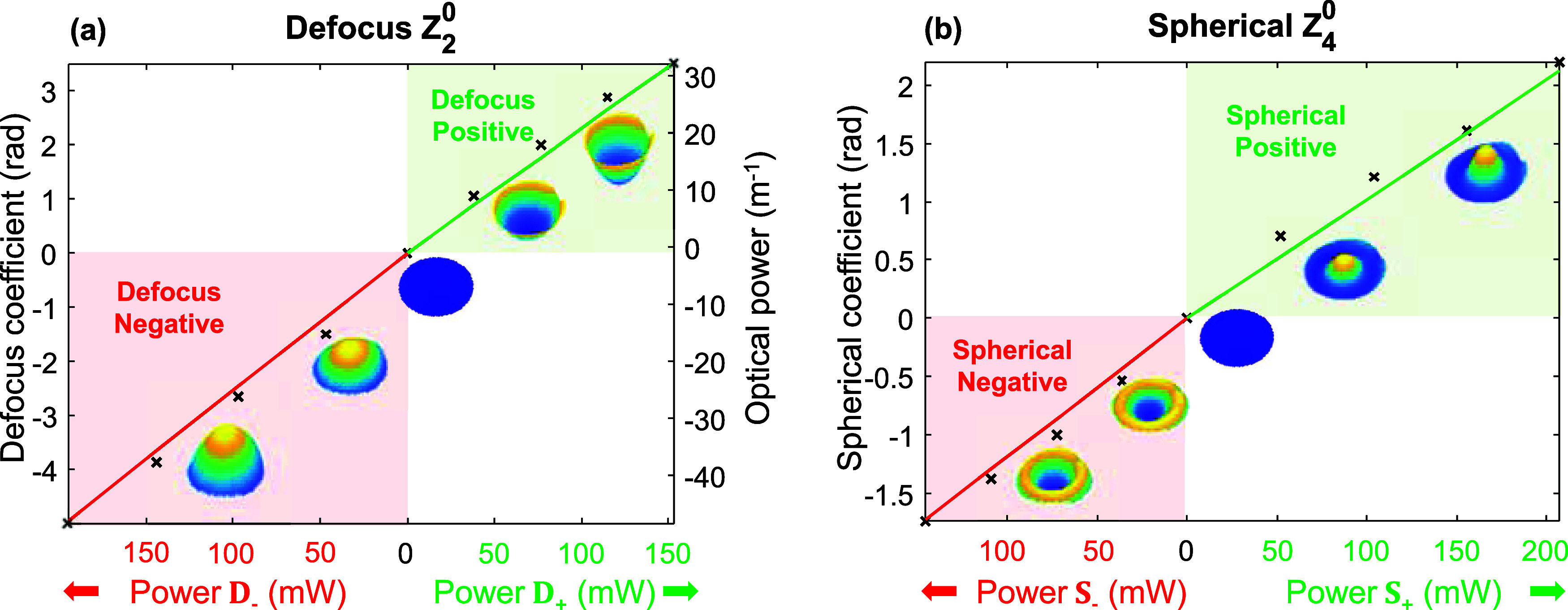
Reconfigurable *SmartLens* dynamic range measured at λ = 605 nm for (a) Defocus *Z*_2_^0^(ρ) and (b) Spherical Aberration *Z*_4_^0^(ρ). In both cases, the values of the Zernike coefficients (vertical axis) are shown against the applied electrical power (horizontal), when applying the appropriate relative weights *D*_–_ and *S*_–_ for negative modes (red curves), or *D*_+_ and *S*_+_ for positive modes (green curves). The insets in (a, b) show 3D representations of the measured wavefronts for various power values. In (a), the right axis indicates the optical power (*P*_op_ = 1/*f*) of the equivalent lens of focal length *f*.

Finally, Figure 4 shows the operational dynamic range of the reconfigurable *SmartLenses* both for defocus *Z*_2_^0^ (see Figure 4a) and spherical aberrations *Z*_4_^0^ (see Figure 4b), measured at λ = 605 nm. In both cases, the optimal power values ***C*** for negative (red curve) and positive (green curve) Zernike coefficients have been applied, while increasing the total power *P*_tot_ (***p*** = *P*_tot_·***C***). For each measurement, the Zernike coefficients of the targeted mode (red bar in Figure 3e) were extracted and reported in Figure 4a,b. To avoid exceeding the PDMS temperature ceiling (*T* = 250 °C),^37^ the applied average voltage was limited to 24 V. In all four cases, a linear response is observed, as expected, enabling continuous independent tuning of both Zernike modes within the ranges of [−5.0 rad, + 3.5 rad] for defocus *Z*_2_^0^ (maximum of 1.8 λ Peak-to-Valley), and [−1.7 rad, + 2.2 rad] for spherical aberration *Z*_4_^0^ (maximum of 1 λ Peak-to-Valley). The right axis in Figure 4a indicates the *SmartLens* optical power *P*_op_ = 1/*f*, calculated from the defocus coefficient α_2_^0^, using the relation . The *SmartLens* can be continuously adjusted from a *P*_op_ = −48.49 m^–1^ diverging lens vergence to a *P*_op_ = 32.09 m^–1^ converging lens. This corresponds to a focal length dynamic range of [*f*_SL_′ = −∞; −21 mm] ∪ [+31 mm; +∞].

## Conclusions

The ability to reconfigure wavefronts in transmission mode is crucial to many applications in the visible, and the *SmartLens* concept can offer a good degree of control with polarization-independent, quasi-achromatic and compact, planar systems. This work shows that even a limited set of resistors (*N* = 4), can be balanced to independently tune pure Zernike modes (*n* = 2) from negative to positive. The linearity of the optical response with electrical power, the absence of coupling between the actuators and the absence of hysteresis (see Supporting S3) arguably make them easier to tune and calibrate than most mechanical-deformation-based systems, which tend to be nonlinear and display hysteresis-related issues. Here, we have shown that a simple calibration step in which only one phase image is acquired for each independent resistor is sufficient to determine the electrical weights to be applied in order to obtain the desired Zernike mode with a chosen amplitude.

Temporally, the response time of these thermal-based devices is driven by the surface area of the resistor.^33^Supporting S4 shows that a 400 μm diameter reconfigurable Smartlens reaches a 10–90% response time of typ. 250 ms. This speed is adapted to e.g., aberration correction in biological media, but smaller resistors can allow faster thermalization. High thermal conductivity transparent materials, as well as overdriving strategies are also interesting possibilities to increase speed.^38,39^ In terms of dynamics, optimizing the magnitude of refractive changes  with reduced power consumption calls for the design of a new generation of materials with large thermo-optical coefficients  in the visible range. We expect this work to foster new material sciences research in this direction.

While more engineered resistors clearly promise more degrees of freedom, including nonsymmetrical Zernike modes or freeform optical functions, an important bottleneck is still the space available to feed these resistors independently in two-dimensional (2D) designs. We expect that the independent control of *n* modes will require *N* = 2*n* resistors since both negative and positive values need to be accessible. This can probably be addressed using 3D designs, i.e., vertically stacked series of reconfigurable *SmartLenses* and/or vertically stacked electrical feeds. Laterally, arranging several of these reconfigurable *SmartLens* systems in arrays also opens new avenues for imaging applications, 3D display and light field imaging. As such, this addressable thermo-optical technology signifies a leap forward toward fully reconfigurable freeform wavefront control.

## Materials and Methods

### *SmartLens* Fabrication

The ITO resistive elements were fabricated using standard UV lithography. In brief, an HMDS monolayer was vapor-phase deposited on a precleaned glass substrate coated with a 40 Ω/sq resistivity ITO layer (CEC040S, PGO-Online GmbH), followed by spin coating a 0.7 μm thick photoresist (AZECI3007) on the top. After a 60 s soft bake at 110 °C and optical lithography exposure for 10 s in hard contact mode, the patterns were postbaked at 110 °C for 60 s before developing them with AZ remover 400 K at 1:4 water mixture for 9 s, followed by 120 s hard bake at 120 °C and 30 s oxygen plasma ashing at 200 W. The ITO layer was etched with HCl(35%)/HNO_3_(65%)/H_2_O = 1:0.08:1 mixture ratio at 33 ± 2 °C for 140 s and rinsed with water before the resist was stripped with acetone and rinsed with IPA. Finally, a *h* = 230 μm thick PDMS membrane was located on top of the heater. The PDMS membrane was prepared by controlled drop-casting using the Sylgard 184 kit at a standard 1:10 ratio. After the cross-linking, the membrane was cut and then attached to the structured ITO by adding a small drop of liquid PDMS in between the membrane and the substrate. Subsequently, the assembled device was baked at 80 °C in a convection oven for 1 h.

### Electronics

The applied voltage is delivered to each actuator of the *SmartLens* by means of Pulse Width Modulation (PWM), in which the duty cycle of a high-frequency square-modulated voltage (0 *V* – *V*_max_ = 60 V) is adjusted in order to produce a time-averaged voltage equivalent to (duty cycle) × *V*_max_. The voltage applied to each resistor is controlled by a PCA9685 12-bit pulse width modulation (PWM) integrated circuit. These systems are driven by a Raspberry 4 computer via a Python script. It allows the control of 16 separate PWM signals at programmable frequencies up to 1526 Hz and duty cycles adjustable from 0 to 100%. These electronics are powered by a 60 V–10 A power supply. Each microresistor (*R*_1–4_) features a resistance of *R_i_* ≈ 10 kΩ.

### Optical and Thermal Characterization

The transmission spectra of the *SmartLenses* were measured by imaging its active area into a fibered spectrometer (Avantes, AvaSpec-ULS2048CL-EVO). The spectrum acquired through *SmartLens* was normalized by the spectrum of the Halogen light source. OPD responses were measured using a custom-made microscope operating in transmission. The *SmartLenses* were illuminated with a halogen lamp, spectrally filtered (λ = 605 nm, Δλ = 55 nm) to avoid coherence artifacts. A Köhler illumination was used and set to illuminate the *SmartLens* with a high spatial coherence (NA_ill_ < 0.1) quasi-plane wave. The *SmartLens* was imaged using a microscope objective (x4, numerical aperture NA = 0.1) on a commercial high-resolution wavefront sensor (SID4, Phasics). The temperature maps were deduced from the OPD images through a deconvolution procedure described in ref (40,41). Briefly, the heat source density map *P*, related to the power dissipated by Joule effect, is first estimated by deconvolving the OPD map with the OPD Green’s function *G*_δ_ and the temperature map is obtained by convolving the power map *P* with the thermal Green’s function *G*_T_: Δ*T* = *P* ⊗ *G*_T_, with, .

## Supporting Information Available

Describes the response of a single thermal-actuator and the minimization of the coupling between actuators; results related to hysteresis and response time are also provided (PDF)
