# Supplementary material for: Reconfigurable Integrated Thermo-Optics for Aberration Correction
Source: ACS Photonics. 2024 Oct 8;11(11):4804–11. doi: 10.1021/acsphotonics.4c01290 (PMC11583300; doi:10.1021/acsphotonics.4c01290)
Supplement: Supplementary file 1 — ph4c01290_si_001.pdf [file ph4c01290_si_001.pdf]

SUPPORTING INFORMATION
Reconfigurable Integrated Thermo-Optics for Aberration Correction

Josep M. Panadés[†], Nadja Rutz[‡], Hadrien M.L. Robert[†], Raphael T. Steffen[‡], Jose García-Guirado[‡], Gilles Tessier[†], Romain Quidant[‡,*], Pascal Berto[†, §, ⱻ,*]

[†]Sorbonne Université, CNRS UMR7210, INSERM UMRS968, Institut de la Vision, Paris, 75012, France.

[‡]Nanophotonic Systems Laboratory, Department of Mechanical and Process Engineering, ETH Zürich, 8092 Zürich, Switzerland.

[§]Université Paris Descartes, Sorbonne Paris Cité, Paris, 75006, France.

[ⱻ]Institut Universitaire de France (IUF), Paris, 75005, France

S1 – Response of a single thermal-actuator

In this section, we evaluate the response of a single thermal actuator to check its linearity with power.

Figure.S1a shows the OPD profile $\delta(P)$ for the outer micro-resistor R_4, measured at different power values P. In order to check the linearity of the OPD response with the applied power, a proportionality factor α and an offset β are estimated for each OPD profile using a non-negative linear least square:

$$\delta(P) = \alpha(P)\cdot\delta(P_{ref}) + \beta(P) \quad \text{(S1)}$$

Each profile is compared to a reference OPD profile $\delta(P_{ref})$ acquired at an electrical power $P_{ref} = 191\ mW$. Using the estimated α and β parameters, the normalized OPD profile can be compared (see Fig S1. b). The superimposed profiles indicate that the wavefront shape does not depend on the applied power. Finally, Figure.S1c shows the evolution of the estimated $\alpha(P)$ values with power when activating each micro-resistor, demonstrating a clear linear behavior for each thermal actuator. In Figure S1d, we also provide, for each thermal actuators, the measured maximum temperature increase depending on the applied power (calculated using a constant resistance value measured for each actuator at ambient temperature).

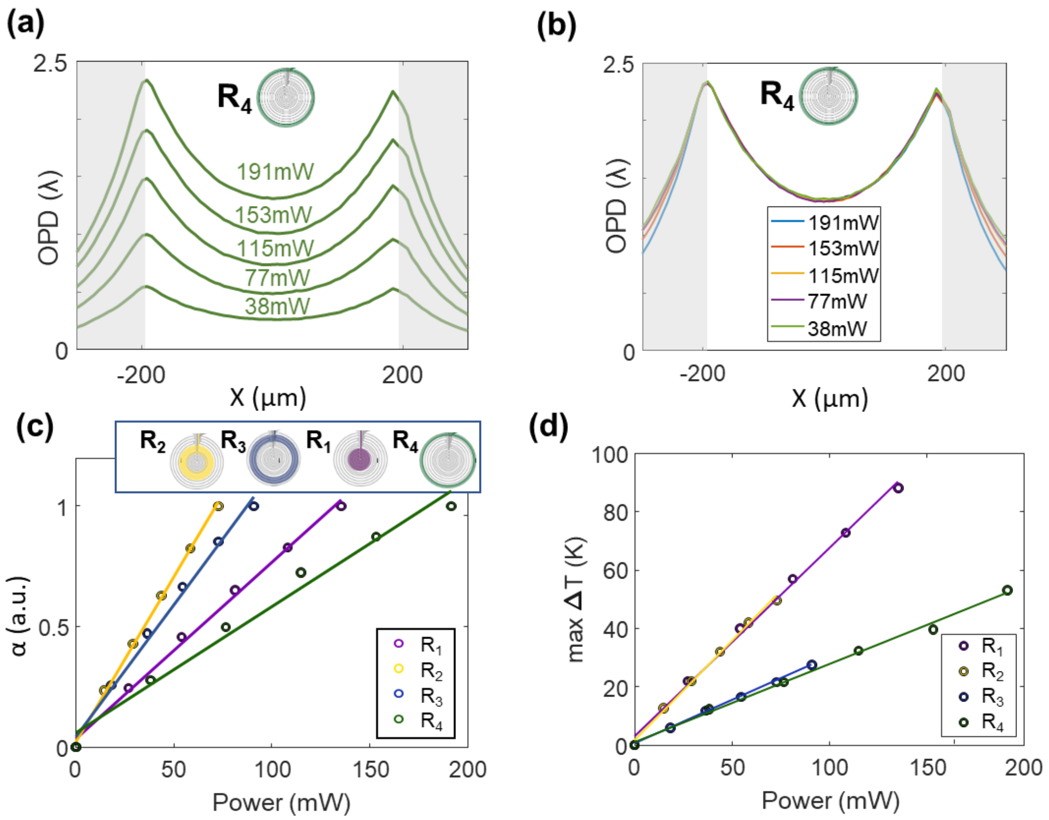

Figure S1: (a) OPD response of microheater 4 at different power values. **(b)** Superimposed responses for the different power values (a proportionality factor and an offset are applied, as defined in Eq. S1). **(c)** Linear behavior of the responses for the different microheaters. **(d)** Temperature dependence of the thermal actuators with applied power, estimated through deconvolution of the OPD images.

S2 – Minimization of the coupling between actuators

This section discusses the electrical coupling between actuators. Figure S2a presents the design of the reconfigurable *SmartLens*, and Figure S2b shows its equivalent electrical circuit.

The feeding and ground electrode design were optimized to create the least amount of spurious aberrations. To this aim, the width of the feeding electrodes was maximised, thus minimizing heat generation outside the resistive heater area. The same applies to the common ground electrode, keeping in mind that it receives current from several resistors and should therefore have a width proportional to the number of resistors. On the other hand, an excessively wide wire leads to a more significant axial symmetry breaking in the OPD map, as notably visible in Figure 1d of the main text. Note that although technologically more complex to fabricate, thicker feed electrodes would allow the reduction of current density while minimizing their surface.

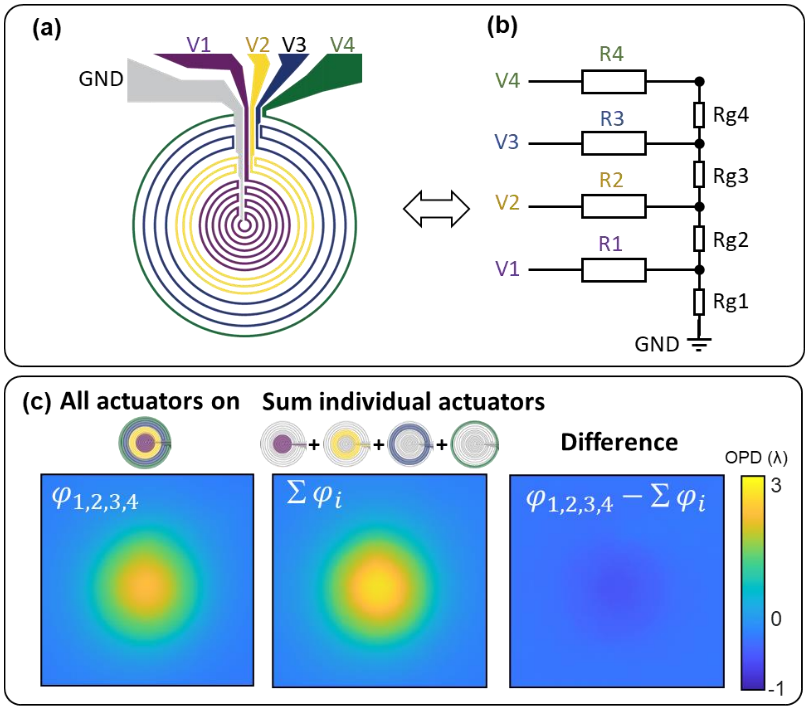

Figure S2: (a) Reconfigurable *SmartLens* design. **(b)** Equivalent electrical circuit of the *SmartLens*. **(c)** OPD maps of all microheaters activated at the same time (φ_{1234}),, sum of individually activated microheaters ($\Sigma\varphi_i$), and their differenceφ_{1234}- $\Sigma\varphi_i$.

Finally, the resistance of the ground wire R_{gi} (Fig. 2Sb) has also been minimized compared to the resistance of the actuators ($R_{gi} \approx R_i/100$) to ensure that the electrical crosstalk (and thus, the thermal and phase crosstalks) between microheaters is minimal since the current tends to circulate more through the lower resistances.

Then, considering that $R_{gi} << R_i$, the voltage present in each resistor R_{gi} can be expressed as:

$$V_{gi} = \frac{R_{gi}}{R_i + \sum_{j=1}^{N} R_{gj}} V_i \; << 0 \qquad (S2)$$

The OPD response when all micro-heaters are activated simultaneously (see Fig. S2c, left) is compared to the sum of the OPD responses when the micro-heaters are independently activated (see Fig. S2c, middle). The negligible difference between these images indicates that there is minimal electrical cross-talk between the thermal actuators, and that the overall response can be assumed to be the sum of the responses from each independently activated heater (see Eq. 5 of the main text).

S3 – Characterization of the hysteresis of the device

This section aims to estimate the hysteresis of the system. To do this, we measured the OPD response of one of the thermal actuators (R_4) during a cycle in which the power applied to the micro-resistor was increased and then decreased. For each state, the measurement was taken more than 0.5 seconds after applying the setpoint, to ensure that the steady-state had been reached (see Supplementary S4). Figure S3 shows the measured OPD in the forward (orange) and backward (blue) directions of the temperature change. The temperature associated with each condition is indicated on the right axis. We observe that the points measured in the forward direction of the temperature change overlap those in the backward direction, indicating that the system does not suffer from hysteresis.

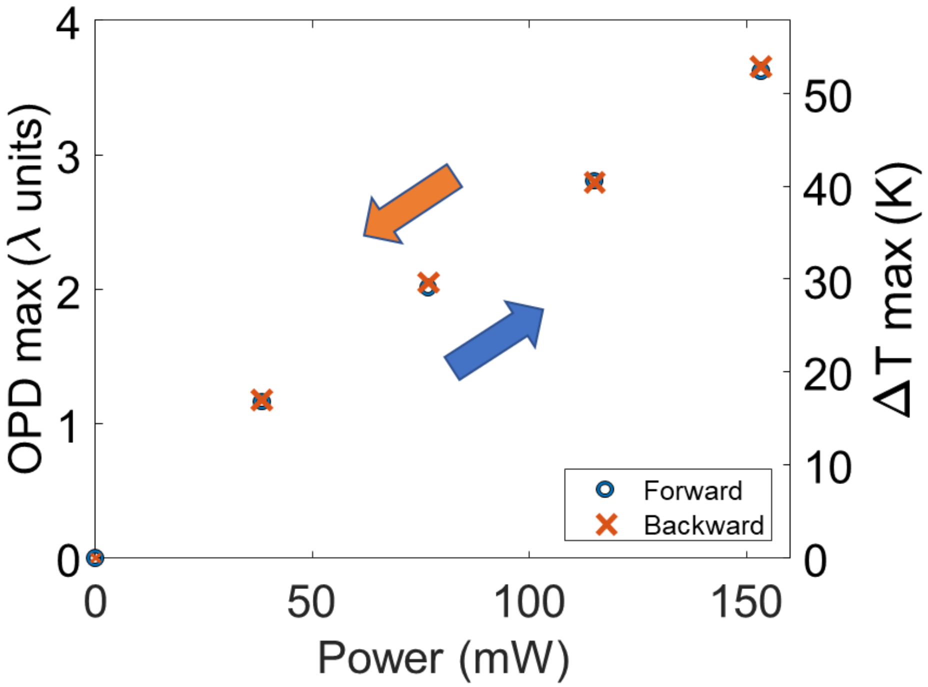

Figure S3: Hysteresis measurement. The measured OPD in the forward (blue circle) and backward (orange cross) directions of the temperature change displays negligible hysteresis.

S4 – Response time

In this section, we evaluate the response time of a 400 µm diameter reconfigurable Smartlens. To achieve this, we simultaneously applied a square modulation to the 4 micro-resistors, at a frequency of f=0.25Hz. The power combination $[p_1\ p_2\ p_3\ p_4]$ applied to each micro-resistor during the "ON state" was chosen to generate a diverging lens ($[p_1\ p_2\ p_3\ p_4] \propto [3\ 3\ 6\ 1]$), corresponding to a total power applied to the Smartlens: $P_{tot} = \sum_{i=1}^{4} p_i = 45\ \mathrm{mW}$. Figure S4a shows the evolution of the measured wavefront over time, from which we estimated the temporal evolution of the Z_2^0 Zernike coefficient for defocus (proportional to the optical power of the generated diverging lens) (see Figure S4b). From this temporal trace, the rise time τ_{rise}= 233 ms and fall time τ_{fall} = 267 ms are extracted by considering the 10% to 90% transition time with respect to the steady-state values. As expected, these response times are of the same order of magnitude as the time scale for heat diffusion[1,2]on a surface with a $D = 400\ \mu m$ diameter: $\tau = D^2/(4a_s) = 120\ \mathrm{ms}$, where a_s is the average thermal diffusivity at the glass/PDMS interface ($a_s \approx 3.4 \cdot 10^{-7}\ \mathrm{m^2.s^{-1}}$).

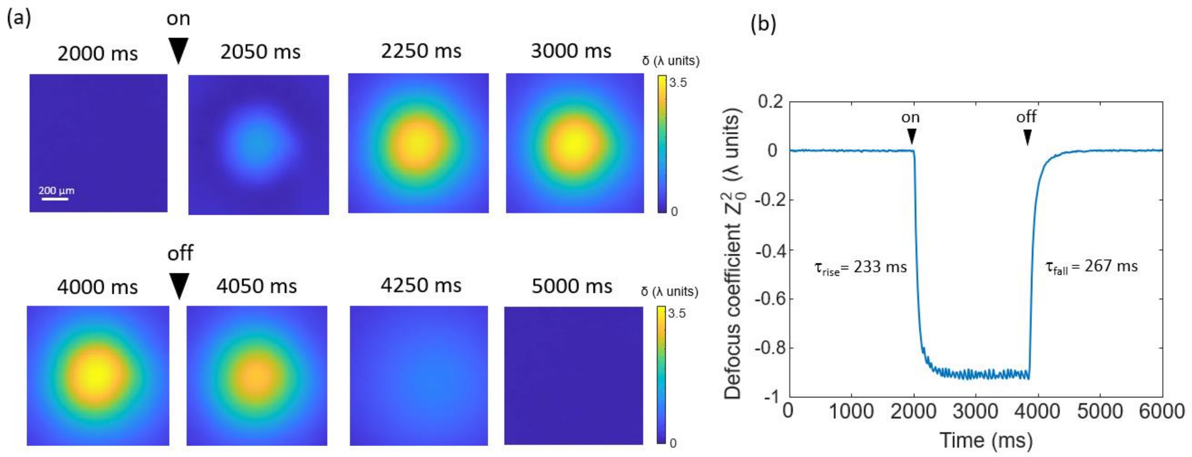

Figure S4: Response time (a) Wavefront images during the transient period when the reconfigurable Smartlens is switched on (top) and switched off (bottom). Here, the power combination ($[p_1\ p_2\ p_3\ p_4] \propto [3\ 3\ 6\ 1]$) applied to each micro-resistor was chosen to generate a diverging lens **(b)** Evolution of the Z_2^0 Zernike coefficient for defocus , from which the rise time (τ_{rise}= 233 ms) and fall time (τ_{fall} = 267 ms) of the device are estimated."

References:

(1) Berto, P.; Mohamed, M. S. A.; Rigneault, H.; Baffou, G. Time-Harmonic Optical Heating of Plasmonic Nanoparticles. *Phys Rev B Condens Matter Mater Phys* **2014**, *90* (3), 035439. https://doi.org/10.1103/PHYSREVB.90.035439/FIGURES/12/MEDIUM.

(2) Berto, P.; Philippet, L.; Osmond, J.; Liu, C. F.; Afridi, A.; Montagut Marques, M.; Molero Agudo, B.; Tessier, G.; Quidant, R. Tunable and Free-Form Planar Optics. *Nature Photonics* **2019** *13:9* 2019, *13* (9), 649–656. https://doi.org/10.1038/s41566-019-0486-3.
